# Supplementary figures and images for: An approach for elucidating dermal fibroblast dedifferentiation in amphibian limb regeneration
Source: Zoological Lett. 2022 Apr 28;8:6. doi: 10.1186/s40851-022-00190-6 (PMC9047331; doi:10.1186/s40851-022-00190-6)

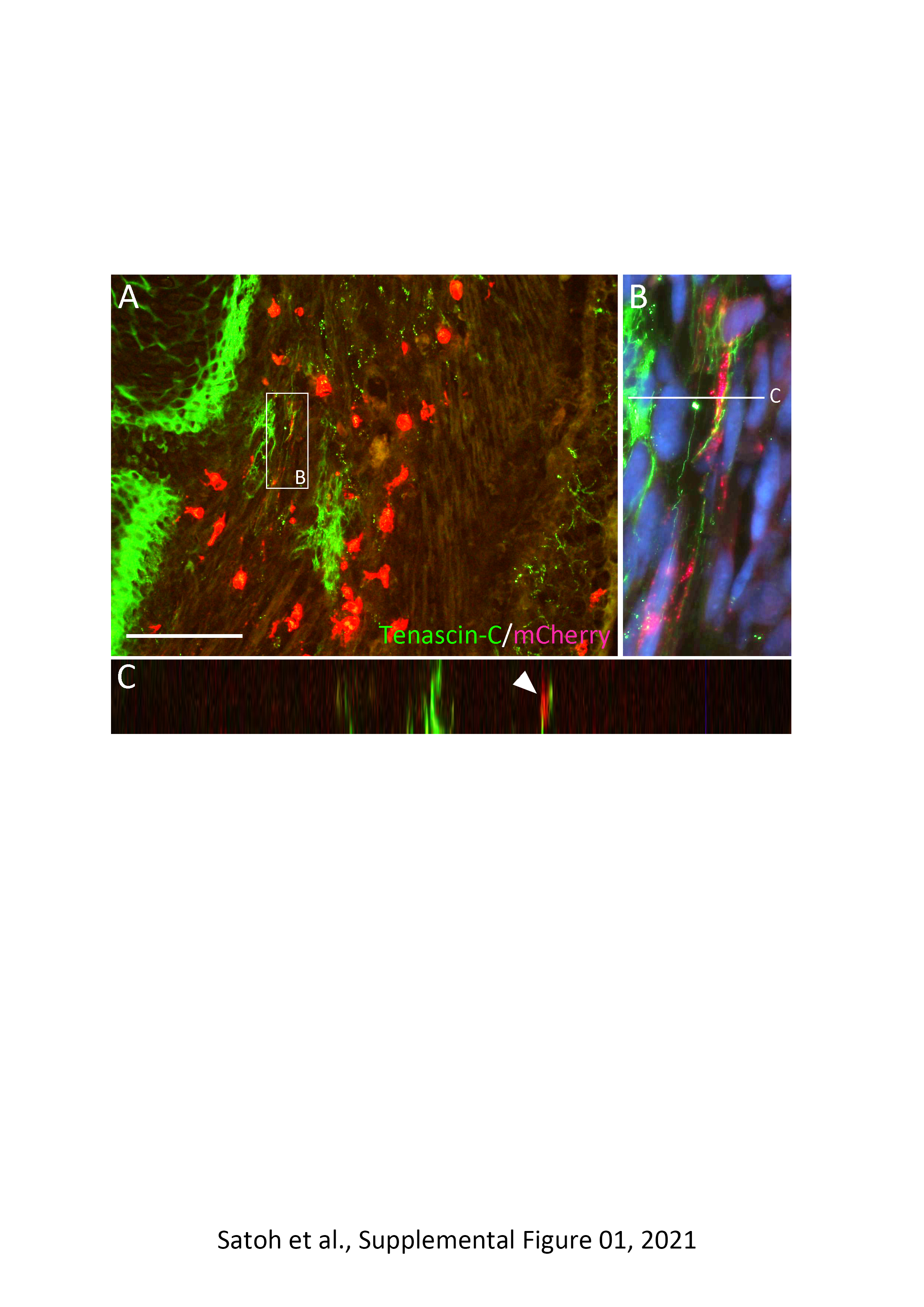

Supplement: Supplementary file 1 — Additional file 1: Supplemental Figure 1. The distribution of the mCherry+ newt cells in the axolotl regenerated limb and Tenascin-C expression. (A) The signals of mCherry and Tenascin-C were visualized by immunofluorescence. The scale bar in A is 200 μm. (B) The higher magnification view of the boxed region in A. The blue color indicates the nuclei. (C) The side view of the region is indicated by the line in B. The Tenascin-C signal is located at the side of the mCherry+ cytoplasm (arrowhead). [file 40851_2022_190_MOESM1_ESM.jpg]

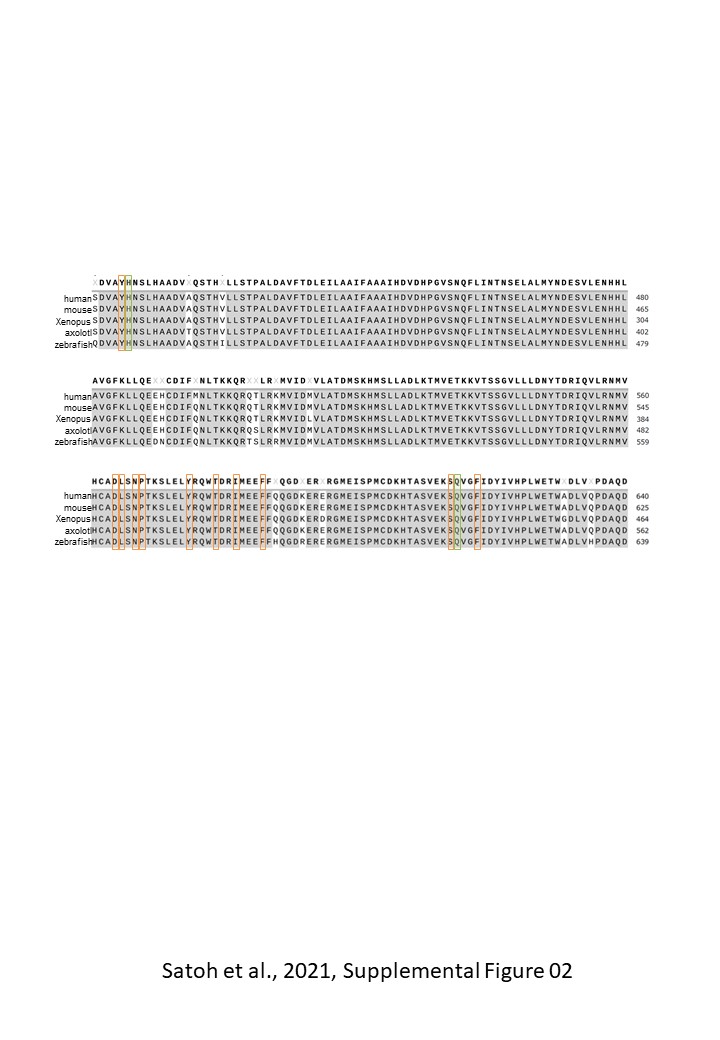

Supplement: Supplementary file 2 — Additional file 2: Supplemental Figure 2. The comparison of the amino acid sequences of PDE4B. The amino acid sequence of PDE4B shown in the figure indicates the nearby region where Rolipram binds. The amino acids surrounded by green lines indicate the residue to which Rolipram forms a hydrogen bond. The amino acid sequence boxed with the orange line indicates the non-ligand residue involved in hydrophobic contact(s). [file 40851_2022_190_MOESM2_ESM.jpg]

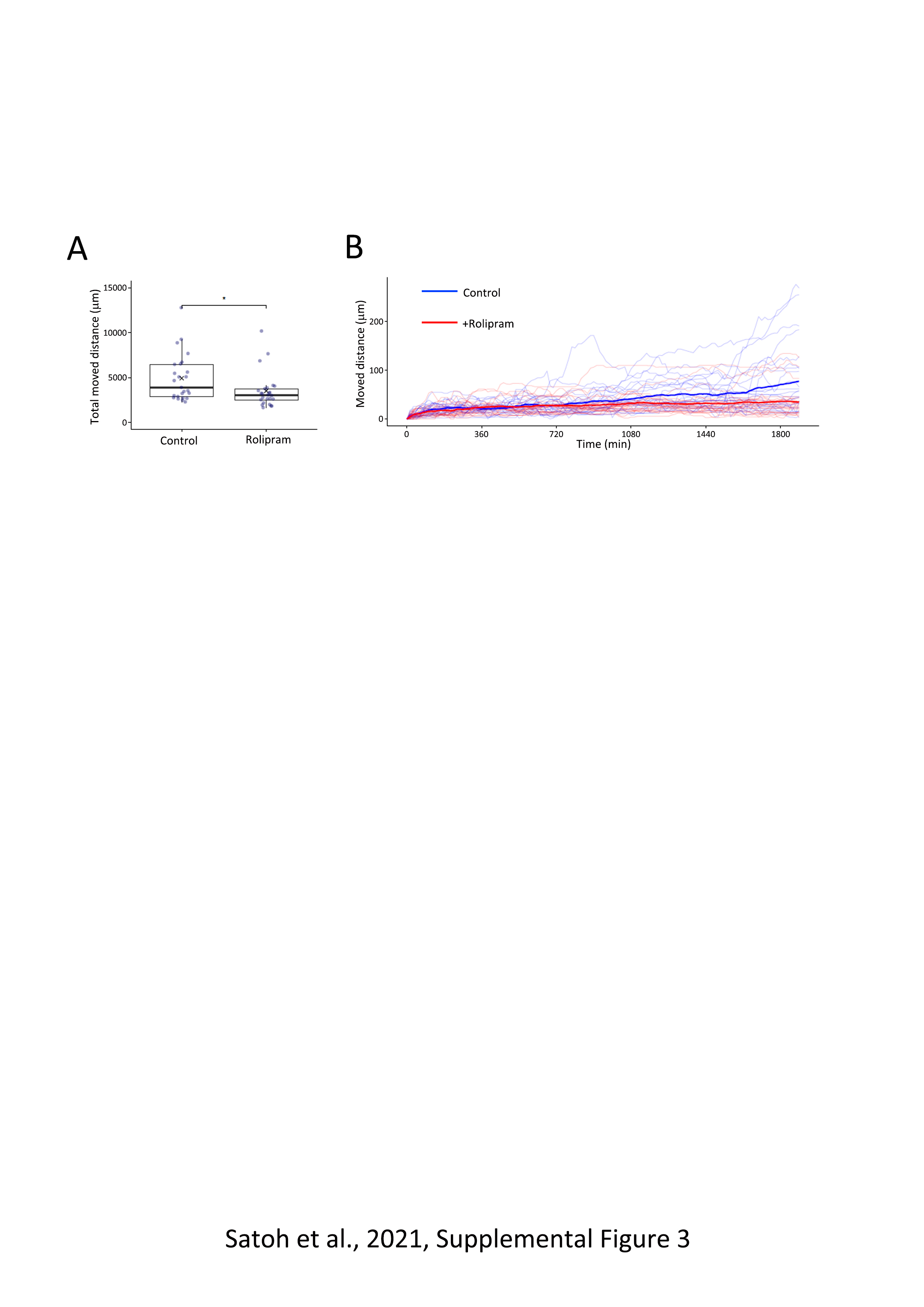

Supplement: Supplementary file 3 — Additional file 3: Supplemental Figure 3. The effect of Rolipram on cell mobility. Cultured newt cells were treated with Rolipram and the cell mobility was traced. (A) The total moved distance was plotted. *p = 0.023. (B) The moved distance and time were shown. The red and blue lines indicate the Rolipram-treated cells and the control cells, respectively. The bold lines show the average. [file 40851_2022_190_MOESM3_ESM.jpg]
